# Facile and Sensitive Electrochemical Sensing of Nitrite in Aquaculture Based on a Fe_3_O_4_/STAB/Chitosan Nanocomposite

**DOI:** 10.3390/nano16140878

**Published:** 2026-07-16

**Authors:** Qihu Dai, Song Zhang, Heng Zhang, Maosai Zhang, Gaoyou Yao

**Affiliations:** 1 School of Fisheries, Xinyang Agriculture and Forestry University, Xinyang 464000, China; 2023210001@xyafu.edu.cn (Q.D.); overlording@126.com (H.Z.); 2 College of Food Science and Technology, Xinyang Agriculture and Forestry University, Xinyang 464000, China; 2024240003@xyafu.edu.cn; 3 School of Artificial Intelligence, Henan Institute of Science and Technology, Xinxiang 453000, China; zhangms@hist.edu.cn

**Keywords:** nitrite, electrochemical sensor, nanocomposite, aquaculture

## Abstract

In this work, an electrochemical sensor based on a Fe_3_O_4_/STAB/chitosan nanocomposite was developed for the detection of nitrite. The nanocomposite exhibited positive charges on its surface, high conductivity, favorable biocompatibility, and strong antifouling ability, enabling electrostatic adsorption of nitrite and thereby enhancing detection sensitivity. A glassy carbon electrode (GCE) modified with this material was employed for nitrite measurement using differential pulse voltammetry (DPV). Under optimized conditions, the Fe_3_O_4_/STAB/chitosan/GCE showed excellent analytical performance for nitrite detection, with a detection limit of 0.151 mg/L. Recovery rates of nitrite in real samples ranged from 95.02% to 107.93%, with relative standard deviations between 0.91% and 5.23%. Moreover, the modified GCE displayed excellent selectivity, reproducibility, and repeatability for nitrite detection. Therefore, this work provides a novel strategy for nitrite monitoring in aquaculture.

## 1. Introduction

Nitrite concentration is widely regarded as a critical indicator of water quality in aquaculture and directly influences aquaculture productivity because of its strong association with fish health and growth performance [1]. In intensive aquaculture systems, high stocking densities and excessive feed inputs promote the accumulation of organic wastes, including uneaten feed and excretory products, which are subsequently degraded by microorganisms, resulting in the release of ammonia as the primary nitrogenous waste product [2,3]. As ammonia concentrations increase, ammonia is oxidized to nitrite by ammonia-oxidizing bacteria (e.g., *Nitrosomonas*), resulting in elevated nitrite levels in the aquatic environment [4]. Nitrite exhibits significant biological toxicity because it can be absorbed into the bloodstream of fish and oxidize hemoglobin into methemoglobin, thereby inducing methemoglobinemia, commonly known as “brown blood disease” [5]. The resulting reduction in the oxygen-carrying capacity of the blood may lead to tissue hypoxia, histopathological damage, decreased appetite, significantly reduced growth performance increased disease susceptibility, and even mortality [6]. The recommended safe concentration of nitrite in aquaculture water is generally considered to be below 0.2 ppm, whereas concentrations exceeding 0.5 ppm are regarded as harmful to most fish species [7]. Therefore, the development of rapid, sensitive, and accurate methods for nitrite monitoring and detection remains a critical challenge in the aquaculture industry.

Numerous analytical techniques are available for nitrite detection, including colorimetry [8], ion chromatography [9], spectrophotometry [10], surface-enhanced Raman spectroscopy [11], and capillary electrophoresis [12]. However, the widespread application of these conventional technologies is limited by their inherent drawbacks, such as time-consuming procedures, complex operational steps, high reagent consumption, long reaction times, and high equipment costs [13]. To meet today’s demands for rapid decision-making, it is necessary to develop an alternative low-cost, reliable, and robust approach for nitrite monitoring in aquaculture.

Electrochemical technology has emerged as a powerful tool for the rapid detection of nitrite, owing to its inherent advantages, including rapid response, high sensitivity, a low detection limit, facile miniaturization and automation, and low cost [14]. To further improve analytical performance, various nanomaterials have been extensively explored as electrode modifiers for the fabrication of electrochemical nitrite sensors [15]. These nanomaterials offer unique morphological features and excellent physicochemical properties, such as high specific surface area, tunable electrocatalytic activity, and synergistic effects of components, which effectively enhance the sensitivity, selectivity, and portability of the sensors [16]. The nanomaterials employed for the modification of bare electrodes are mainly metal nanoparticles (e.g., Au, Ag) [17,18], metal oxides (e.g., ZnO, Fe_3_O_4_) [19,20], and carbon-based nanomaterials (e.g., graphene, carbon nanotubes, carbon nanofibers) [21,22,23].

Chitosan (CS), a naturally occurring polysaccharide derived from chitin via deacetylation, is widely recognized as an excellent biopolymer for electrochemical sensing applications due to its unique physicochemical attributes, including excellent film-forming ability, biocompatibility, and abundant reactive functional groups [24,25,26,27]. In particular, the primary amino and hydroxyl functional groups on its macromolecular backbone not only enable straightforward chemical modification but also facilitate the preconcentration of target analytes via hydrogen bonding and electrostatic interactions [26,27,28]. Furthermore, chitosan possesses the ability to enhance charge transfer kinetics at the interface of electrode and electrolyte, offering a versatile platform for constructing modified electrodes with improved analytical performance [27,29]. Moreover, the combination of chitosan with various nanomaterials such as metal oxides, carbon-based materials, and cationic surfactants has been shown to significantly enhance the sensitivity, selectivity, and stability of electrochemical sensing systems [9,30,31,32].

Stearyltrimethylammonium bromide (STAB) is a quaternary ammonium cationic surfactant featuring a hydrophilic headgroup and a hydrophobic tail. In composite material systems, STAB serves as an effective dispersant that prevents nanoparticle agglomeration and imparts a positive charge to the composite, thereby facilitating the adsorption of nitrite anions [32,33]. When immobilized onto an electrode surface, STAB forms a supramolecular structure that lowers the electrode surface energy, thereby promoting the mass transfer of target analytes toward the electrode surface and consequently enhances the electrochemical sensing performance [32,34].

To the best of our knowledge, no electrochemical sensor based on the ternary composite of Fe_3_O_4_ nanoparticles, CS, and STAB has been reported for the sensitive detection of nitrite. In this work, a simple, efficient, and sensitive electrochemical method for nitrite determination in aquaculture water samples is proposed by modifying a GCE with this novel composite. The specific objectives of this investigation are: (1) to prepare and characterize the Fe_3_O_4_/STAB/CS nanocomposite; (2) to evaluate the electrochemical performance of the Fe_3_O_4_/STAB/CS modified GCE by cyclic voltammetry (CV) and differential pulse voltammetry (DPV); and (3) to investigate the selectivity, reproducibility, and repeatability of the Fe_3_O_4_/STAB/CS/GCE.

## 2. Materials and Methods

### 2.1. Materials and Apparatus

CS with a degree of deacetylation of 85% and STAB were supplied by Runyou Chemical Co., Ltd. (Shenzhen, Guangdong, China). Ferroferric oxide (Fe_3_O_4_), potassium chloride (KCl), disodium hydrogen phosphate dodecahydrate (Na_2_HPO_4_·12H_2_O), sodium dihydrogen phosphate dihydrate (NaH_2_PO_4_·2H_2_O), and agar powder were purchased from Tianjin Zhonglian Chemical Reagent Co., Ltd. (Tianjin, China). Sodium nitrite (NaNO_2_) was obtained from Shanghai Macklin Biochemical Technology Co., Ltd. (Shanghai, China). Cadmium chloride (CdCl_2_), zinc sulfate (ZnSO_4_), manganese (II) chloride (MnCl_2_), glacial acetic acid (CH_3_COOH), magnesium chloride (MgCl_2_), copper (II) sulfate (CuSO_4_), and sodium bicarbonate (NaHCO_3_) were obtained from Sangon Biotech Co., Ltd. (Shanghai, China). A series of 0.1 mol/L phosphate buffer solutions (PBS, pH = 7.0) with varying nitrite concentrations was prepared from NaH_2_PO_4_·2H_2_O and Na_2_HPO_4_·12H_2_O.

A CHI660F electrochemical workstation purchased from Shanghai Chenhua Instrument Co., Ltd. (Shanghai, China) was used to perform the electrochemical tests. A three-electrode system was adopted, in which a GCE (diameter = 3 mm) produced by Shanghai Ciyue Industrial Co., Ltd. (Shanghai, China) served as the working electrode, a saturated calomel electrode (SCE) fabricated by Shanghai Chenhua Instrument Co., Ltd. was used as the reference electrode, and a platinum wire manufactured by Shanghai Sanmusk Industry Co., Ltd. (Shanghai, China) was employed as the auxiliary electrode. The morphology of the Fe_3_O_4_/STAB/CS nanocomposite was characterized using a scanning electron microscope (SEM; Sigma 360, ZEISS, Oberkochen, Baden-Württemberg, Germany) by Shiyanjia Lab. The prepared material was characterized by Fourier transform infrared spectrometer (FTIR) (Nicolet iS20, Thermo Fisher Scientific, Waltham, MA, USA) in a range of 400–4000 cm^−1^. The crystal structure of the prepared material was characterized by Shiyanjia Lab using X-ray diffractometer (XRD) (SmartLab SE, Rigaku, Akishima-shi, Tokyo, Japan). The surface elemental composition and chemical states of the prepared materials were characterized by Shiyanjia Lab using X-ray photoelectron spectrometer (XPS) (ESCALAB QXi, Thermo Fisher Scientific, East Grinstead, West Sussex, UK). A 101-0B electric thermostatic blast drying oven (Yixin Scientific Instruments Co., Ltd., Shanghai, China) was used as a dryer to prepare the needed electrode nanomaterials.

### 2.2. Preparation of Various Modified GCEs

Briefly, 2.0 mg of Fe_3_O_4_ and 1.5 mg of STAB were added into a 10 mL centrifuge tube, followed by the addition of 1 mL of 0.01 wt% CS solution. The resulting dispersion was then sonicated for 10 min to ensure uniform mixing. Subsequently, 7 μL of the aforementioned mixture was drop-cast onto the surface of the GCE, and the electrode was dried in a thermostatic drying oven. Finally, the Fe_3_O_4_/STAB/CS/GCE was successfully obtained once the electrode surface was completely dried. For comparison purposes, Fe_3_O_4_, and Fe_3_O_4_/CS were also separately immobilized onto the GCE surfaces following the same experimental procedure.

### 2.3. Electrochemical Measurement Conditions

The DPV and CV were applied to characterize various modified GCEs in 0.1 mol/L PBS (pH = 7.0) containing different nitrite concentrations at 25 °C. For the DPV technique, the operating parameters were set as follows: pulse width of 0.2 s, potential increment of 0.005 V, quiet time of 5 s, sample width of 0.0167 s, pulse period of 0.5 s, pulse amplitude of 0.025 V, and potential range of 0.4 V to 1.2 V. For the CV technique, the quiet time was set to 2 s, the potential range was 0.2 V to 1.4 V, and the sample interval was 0.001 V.

### 2.4. Real Water Samples Testing

To assess the practical applicability of the sensor for nitrite detection, water samples were collected from fish and shrimp holding tanks referred to as S_1_ and S_2_, respectively, at Xinyang Agriculture and Forestry University. After filtration through a 0.22 µm membrane to eliminate impurities, the samples were stored at 4 °C for subsequent use.

### 2.5. Statistical Analysis

The data were plotted using Origin 2026 (OriginLab Co., Ltd., Northampton, MA, USA). The linear regression equation and correlation coefficient (R^2^) between the nitrite concentration and oxidation peak current were obtained from the linear fitting using Origin 2026.

## 3. Results and Discussion

### 3.1. Preparation and Sensing Principle of the Fe_3_O_4_/STAB/CS/GCE

**Scheme 1:**
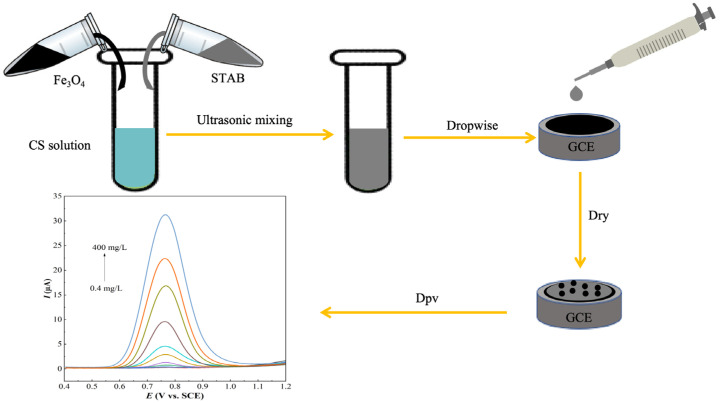
Overview of Fe_3_O_4_/STAB/CS/GCE preparation and the nitrite electrochemical detection.

A schematic diagram illustrating the preparation of Fe_3_O_4_/STAB/CS/GCE and the electrochemical detection of nitrite is presented in Scheme 1. Firstly, Fe_3_O_4_ and STAB were dispersed within a CS solution to prepare the composite-modified electrode. Specifically, STAB introduced a positively charged interface, which strengthened the affinity between the electrode surface and the target analytes. At the same time, CS was utilized as a stable supporting matrix as a result of its excellent film-forming properties, biocompatibility, and good adsorption performance [24,25]. The constructed Fe_3_O_4_/STAB/CS/GCE could attract negatively charged nitrite ions via electrostatic interactions, and also could facilitate electron transfer processes, thereby enhancing the overall sensitivity of nitrite detection. Previous studies have demonstrated that nanomaterials with a positively charged surface could adsorb negatively charged nitrite ions via electrostatic interactions [32].

### 3.2. Characterization of the Fe_3_O_4_/STAB/CS

**Figure 1:**
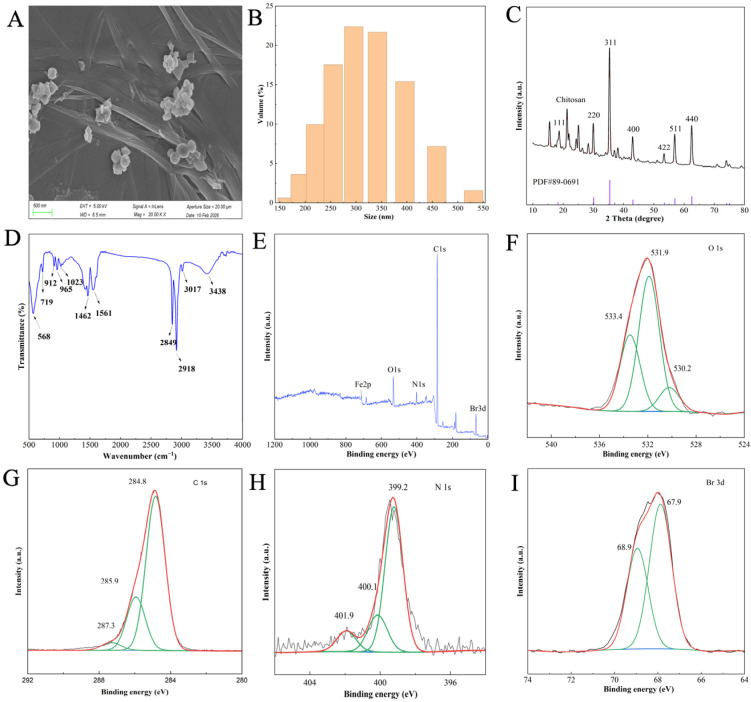
(**A**) SEM image of Fe_3_O_4_/STAB/CS, (**B**) Dynamic light scattering of Fe_3_O_4_, (**C**) XRD of Fe_3_O_4_/STAB/CS, (**D**) FTIR of Fe_3_O_4_/STAB/CS, (**E**) XPS survey spectrum of Fe_3_O_4_/STAB/CS, (**F**) O 1s spectrum, (**G**) C 1s spectrum, (**H**) N 1s spectrum, and (**I**) Br 3d spectrum.

SEM, DLS, XRD, FTIR, and XPS analyses were performed to characterize the Fe_3_O_4_/STAB/CS composite (Figure 1). The composite exhibited a continuous and wrinkled CS film matrix under SEM (Figure 1A). Sphere-like Fe_3_O_4_ nanoparticles were distributed on the membrane surface without severe agglomeration. In Figure 1B, the particle sizes of the Fe_3_O_4_ ranged from 164 to 531 nm. The heterogeneous particle size phenomenon have also been verified by other researchers [35]. A characteristic peak was observed at about 2*θ* = 21.8° in the XRD pattern (Figure 1C), which is characteristic of CS [36]. In addition, distinct diffraction peaks located at 18.3°, 30.1°, 35.5°, 43.1°, 53.5°, 57.0° and 62.6° are assigned to the (111), (220), (311), (400), (422), (511), and (440) crystal planes of Fe_3_O_4_, respectively, which are consistent with the standard spinel Fe_3_O_4_ reference (PDF# No. 89-0691) [37].

The FTIR spectrum of the composite showed a sharp peak assigned to the Fe-O stretching at 569 cm^−1^ (Figure 1D), which corroborates the iron oxide nature of the nanomaterials [38]. The weak band near 913 cm^−1^ is generally assigned to the C-O-C bridge and glucosidic linkage of amides [39]. Peaks at 1023, 1561, and 3438 cm^−1^ were assigned to the C-O stretching vibrations, N-H bending, and O-H/N-H stretching vibrations of CS, respectively [36,40,41]. The absorption bands at 2849 and 2918 cm^−1^ were attributed to the C-H stretching of the long alkyl chains in CS and STAB [41,42,43]. As presented in Figure 1E, the survey XPS spectrum of the Fe_3_O_4_/STAB/CS composite displayed characteristic signals of C, N, O, and Fe elements. The O 1s peak at 530.2 eV (Figure 1F) was assigned to the Fe-O bond of Fe_3_O_4_ [44]. The peaks at 531.9 eV and 533.4 eV were assigned to the N-C=O and C-O-C/C-OH bonds of CS, respectively [45]. As shown in Figure 1G, the C 1s peak at 284.8 eV was correspond to C-C/C-H, originating from the aliphatic backbones of both CS and STAB [46]. The peaks at 285.9 was correspond to C-OH/C-NH_2_/C-O-C bonds of CS and quaternary ammonium carbon of STAB [47,48]. The peak at 287.3 eV was correspond to N-C=O bond of CS [47]. In Figure 1H, the N 1s peaks at 399.2 eV and 400.1 eV were correspond to the amino nitrogen and amide nitrogen of CS, respectively [49,50]. Additionally, a peak at 401.9 eV was attributed to the quaternary ammonium nitrogen of STAB, consistent with the typical binding energy values reported for quaternary ammonium compounds [51]. In Figure 1I, the Br 3d peaks at 67.9 eV and 68.9 eV were identified as the characteristic spin–orbit splitting peaks of Br^−^ in STAB [52]. Based on the comprehensive characterization results from SEM, XRD, FTIR, and XPS, it can be confirmed that the Fe_3_O_4_/STAB/CS composite was successfully fabricated.

### 3.3. Electrochemical Behaviors of Different Modified GCEs

**Figure 2:**
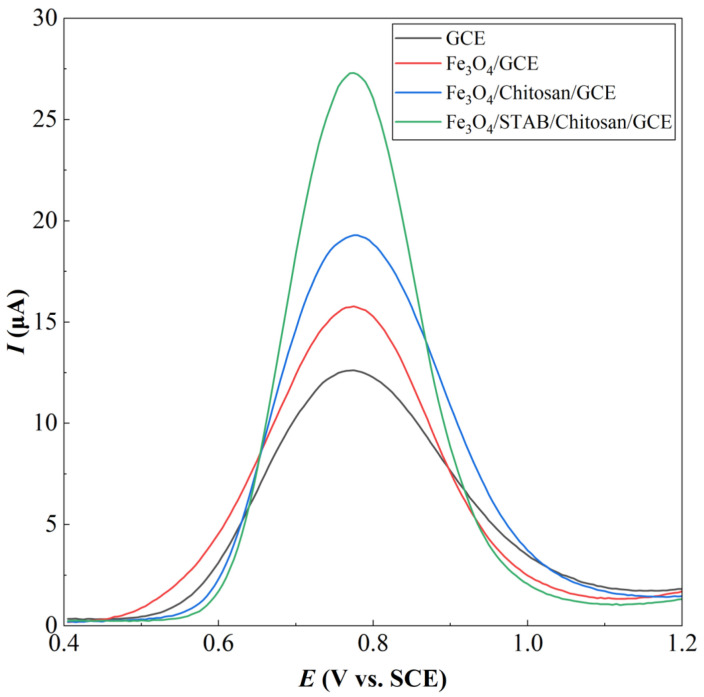
DPV curves of different modified GCEs for nitrite detection containing 400 mg/L nitrite PBS (pH = 7.0).

DPV was used to investigate the electrochemical response of nitrite (400 mg/L) in PBS (pH = 7.0) at various modified GCEs (Figure 2). As illustrated in Figure 2, all electrodes exhibited an anodic oxidation peak of nitrite at approximately +0.76 V, a finding consistent with previous reports [53]. When the bare GCE was modified with Fe_3_O_4_, the peak current went up from 12.6 μA to 15.8 μA, which could be attributed to the generation of additional active sites and the facilitation of electron transfer by Fe_3_O_4_. In particular, Fe_3_O_4_ nanoparticles have been reported to provide abundant active sites and serve as an efficient electron mediator [54,55].

The Fe_3_O_4_-modified electrode exhibited a significantly higher peak current than the bare GCE. This enhanced electrochemical response can be primarily attributed to the excellent electron transfer capability of the Fe_3_O_4_ nanoparticles. This capability arises from the mixed-valence nature of Fe_3_O_4_, which contains both Fe^2+^ and Fe^3+^ ions, enabling efficient electron hopping between these oxidation states and thereby facilitating the redox reaction of nitrite at the electrode surface [56,57].

The electrode coated with Fe_3_O_4_/CS showed a higher peak current compared to that of the electrode coated with Fe_3_O_4_. A plausible explanation is that chitosan formed a uniform, hydrophilic film on the electrode surface [58]. The abundant amine and hydroxyl functional groups on the chitosan backbone can serve as active sites for analyte immobilization and promote charge transfer at the electrode, thereby enhancing the overall electrochemical performance [59]. The electrode coated with Fe_3_O_4_/STAB/CS showed a higher peak current compared to that of the electrode coated with Fe_3_O_4_/CS, owing to the electrostatic enrichment of target molecules and a concurrent reduction in interfacial impedance [32,60]. STAB can prevent nanoparticle aggregation through its dispersing ability and impart a positive surface charge to the composite, thereby facilitating the electrostatic attraction of nitrite anions [32,33]. Moreover, the positively charged STAB layer is believed to reduce the electrostatic repulsion and promote the enrichment of nitrite toward the electrode surface, leading to an enhanced oxidation peak current [61]. Therefore, Fe_3_O_4_/STAB/CS modified GCE could be used for sensitive nitrite detection.

### 3.4. Effect of Scan Rate, STAB Concentration and Drop-Casting Volume

**Figure 3:**
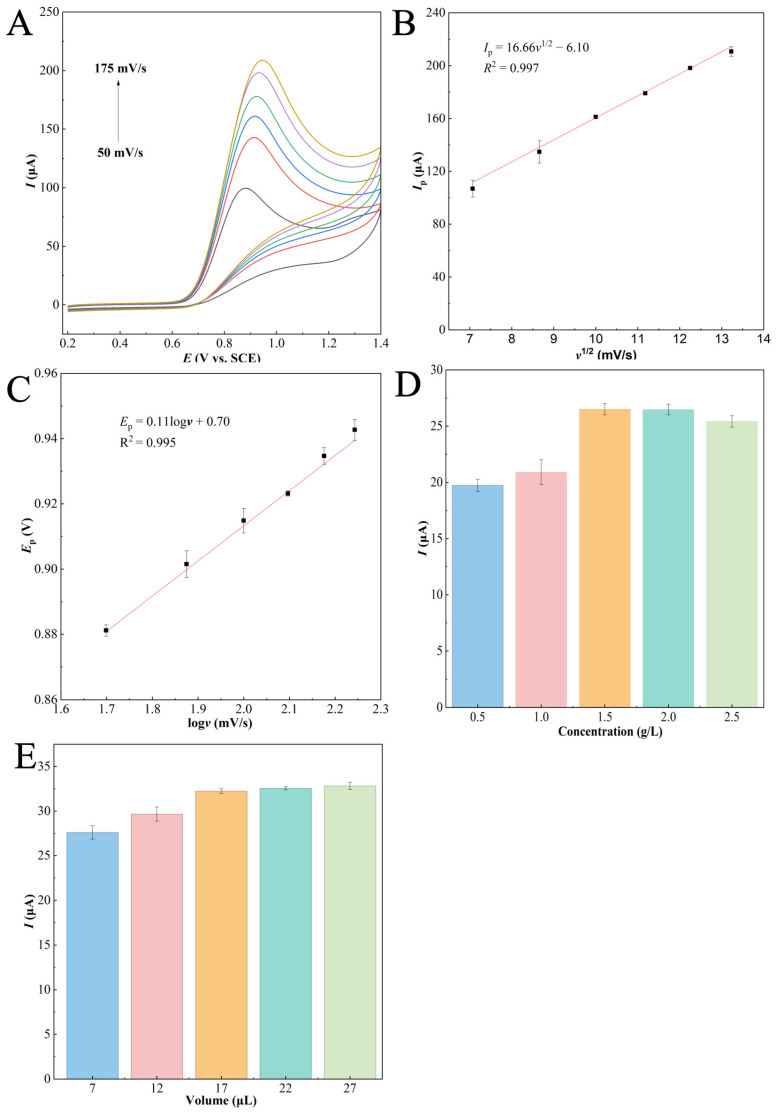
(**A**) CV curves of nitrite for Fe_3_O_4_/STAB/CS/GCE at various scan rates (*v*) containing 400 mg/L nitrite PBS (pH = 7.0), (**B**) the relationship between the square root of scan rate (*v*^1/2^) and the oxidation current (*I*_p_) of nitrite, (**C**) the relationship between the logarithm of scan rate (log*v*) and the oxidation peak potential (*E*_p_) of nitrite, (**D**) Effect of STAB concentration on the oxidation current of nitrite at Fe_3_O_4_/STAB/CS/GCE containing 400 mg/L nitrite PBS (pH = 7.0), (**E**) Effect of drop-casting volume on the oxidation current of nitrite at Fe_3_O_4_/STAB/CS/GCE containing 400 mg/L nitrite PBS (pH = 7.0).

The scan rate, STAB concentration and drop-casting volume are widely recognized as key factors influencing the electrochemical detection performance. Accordingly, the influence of scan rate on the nitrite response current was investigated using CV at the Fe_3_O_4_/STAB/CS modified GCE in PBS containing 400 mg/L nitrite (Figure 3). As shown in Figure 3A, the anodic peak current intensity for nitrite gradually increased with the increase in scan rates ranging from 50 mV/s to 175 mV/s. As shown in Figure 3B, a positive correlation was observed between the oxidation current (*I*_p_) of nitrite and the square root of the scan rate (*v*^1/2^), and the corresponding linear regression equation was expressed as Equation (1):

*I*_p_ (μA) = 16.66 *v*^1/2^ − 6.10 (R^2^ = 0.997) (1)

Furthermore, as depicted in Figure 3C, the anodic peak potential (*E*_p_) of nitrite shifted positively as the logarithm of scan rate (log*v*) increased. The corresponding linear regression equation was given by Equation (2):

*E*_p_ (V) = 0.11 log*v* + 0.70 (*R*^2^ = 0.995) (2)

Based on the above-mentioned results, the oxidation process of nitrite at the Fe_3_O_4_/STAB/CS modified GCE is a diffusion-controlled and irreversible process [62,63]. The number of electrons transferred during nitrite oxidation could be calculated by means of Equation (3) [32]:

*E*_p_ = 2.303 *RT* [*n*(1 − *α*) *F*]^−1^ log*v* + *E*^0′^ (3)

In this equation, *E*^0′^ represents the formal potential, *T* stands for the temperature in Kelvin (298 K), *R* denotes the gas constant (8.314 J/(mol·K)), *F* is the Faraday constant (96,480 C/mol), *α* refers to the electron transfer coefficient, and *n* is the number of electrons transferred. For irreversible electrochemical reactions, the value of *α* is typically taken as 0.5 [32]. Under this premise, the calculated results revealed that the electrochemical oxidation of nitrite proceeded through a single-electron transfer process as the rate-determining step, which aligns well with the conclusions reported in a previous study [64]. The oxidation of NO_2_^−^ on Fe_3_O_4_/STAB/CS/GCE can be described as follows [64]

NO_2_^−^ → NO_2_ + e^−^ (4)

Figure 3D illustrated the effect of different STAB concentrations on the peak current of nitrite. As the STAB concentration increased from 0.5 to 1.5 g/L, the peak current rose accordingly, while a further increase to 2.5 g/L led to a plateau in the current response. A similar concentration-dependent behavior has been reported for STAB-modified electrodes in the electrochemical detection of methotrexate, where the peak current increased with STAB concentration up to an optimum point and then stabilized [60]. Therefore, the optimal STAB concentration for achieving the best current signal was determined to be 1.5 g/L.

As shown in Figure 3E, the peak current of nitrite gradually increased as the drop-casting volume increased from 7 μL to 17 μL, due to enhanced electrostatic accumulation of nitrite anions, as evidenced by analogous studies on chitosan and cationic surfactant-modified sensors [30]. However, beyond an optimal volume, the response current reached a plateau, which can be explained by the saturation of active sites on the electrode surface and an increased diffusion barrier from an overly thick modification layer. Such a trend of signal saturation or slight decay after exceeding the optimal modifier amount has been well documented in a previous study [65].

### 3.5. Detection of Nitrite

**Figure 4:**
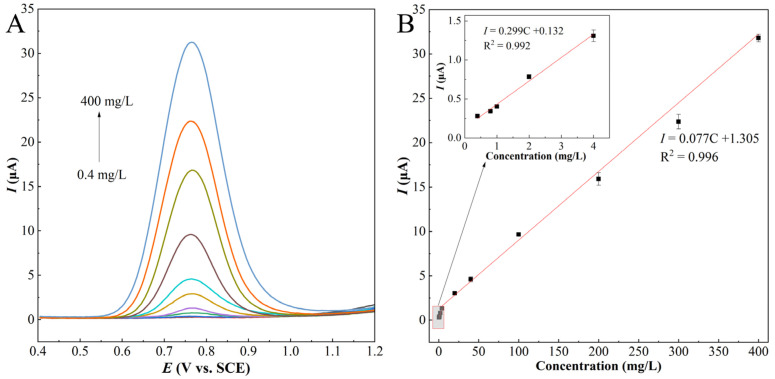
(**A**) The DPV curves of Fe_3_O_4_/STAB/CS/GCE containing various concentrations of nitrite PBS (pH = 7.0), (**B**) the calibration curve between anodic oxidation currents and concentrations of nitrite.

Under the optimized conditions, the electrochemical detection performance of the Fe_3_O_4_/STAB/CS/GCE for nitrite was evaluated using DPV in PBS containing various concentrations of nitrite. The obtained DPV and corresponding calibration curves are presented in Figure 4. As illustrated in Figure 4A, the oxidation peak current of nitrite steadily increased as its concentration rose from 0.4 mg/L to 400 mg/L. Afterwards, two linear relationships could be observed by drawing the standard curves in Figure 4B. The corresponding linear regression equations were *I* (μA) = 0.299C + 0.132 (R^2^ = 0.992) in the range of 0.4 mg/L to 4 mg/L and *I* (μA) = 0.077C +1.305 (R^2^ = 0.996) in the range of 4 mg/L to 400 mg/L.

For the proposed nitrite electrochemical sensor, the limit of detection (LOD) was calculated to be 0.151 mg/L (S/N = 3). This value was below the recommended safe limit, demonstrating the sensor’s practical applicability for aquaculture water quality monitoring [7]. Moreover, the detection capability of this sensor was compared with that of other previously reported nitrite electrochemical sensors, and the comparative data are presented in Table 1. The sensor developed in this work demonstrated a superior detection limit or linear range relative to those reported in previous study (Table 1).

### 3.6. Selectivity, Reproducibility, and Repeatability

**Figure 5:**
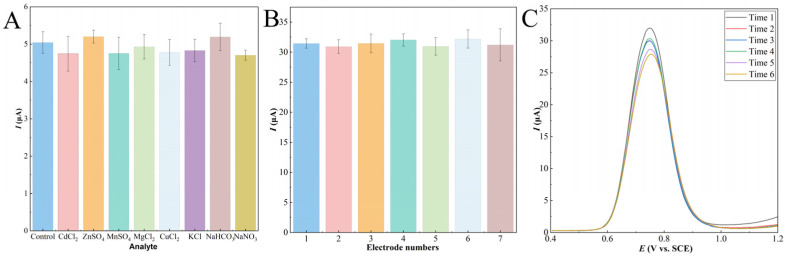
(**A**) Selectivity of Fe_3_O_4_/STAB/CS/GCE containing 40 mg/L nitrite PBS (pH = 7.0), (**B**) reproducibility of Fe_3_O_4_/STAB/CS/GCE for six parallel electrodes containing 400 mg/L nitrite PBS (pH = 7.0), (**C**) repeatability of Fe_3_O_4_/STAB/CS/GCE for six consecutive measurements (single electrode) containing 400 mg/L nitrite PBS (pH = 7.0).

Selectivity, reproducibility, and repeatability (Figure 5) are key performance metrics for the Fe_3_O_4_/STAB/CS modified GCE. These characteristics were systematically evaluated using DPV in PBS (pH = 7.0). As depicted in Figure 5A, the DPV oxidation current of nitrite (40 mg/L in PBS, pH 7.0) remained nearly unchanged upon individual addition of 400 mg/L each of CaCl_2_, ZnSO_4_, MnCl_2_, MgCl_2_, CuSO_4_, KCl, and NaNO_3_. The maximum variation in the nitrite oxidation current was 6.30% relative to the current measured without interferents, indicating that the proposed sensor possesses strong anti-interference ability and high selectivity. Reproducibility was assessed using six independently fabricated electrodes under identical conditions in PBS containing 400 mg/L nitrite (Figure 5B). The relative standard deviation (RSD) of the DPV oxidation currents was 1.58%, demonstrating that the proposed electrochemical sensor exhibits good reproducibility. Repeatability was evaluated by performing six consecutive measurements on a single electrode under the same conditions (Figure 5C). The RSD of the DPV oxidation currents was 4.74%, indicating the sensor’s good repeatability [71,72].

### 3.7. Detection of Nitrite in Real Water Samples

To evaluate the practical feasibility of the Fe_3_O_4_/STAB/CS modified GCE for nitrite detection, recovery tests were conducted on pretreated water samples. Different concentrations of nitrite were spiked into the water samples, and each spiked sample was measured in triplicate (*n* = 3) using the proposed electrochemical sensor. As summarized in Table 2, the recoveries obtained for nitrite detection ranged from 95.02% to 107.93%, indicating satisfactory trueness. The recovery determination itself carries an intrinsic uncertainty, due to potential matrix effects [73]. Therefore, the RSD values were incorporated into the overall measurement uncertainty budget to enhance result reliability. The relative standard deviations (RSDs) were between 0.91% and 5.23%, demonstrating good precision. These results demonstrate that the Fe_3_O_4_/STAB/CS modified GCE provides acceptable accuracy and precision for nitrite determination in real water samples, thereby confirming its potential for routine food safety analysis.

## 4. Conclusions

A facile method was employed to prepare a Fe_3_O_4_/STAB/CS composite, which was subsequently used to fabricate an electrochemical sensor for nitrite detection. The incorporation of Fe_3_O_4_/STAB/CS significantly enhanced the sensor’s sensitivity toward nitrite. Under optimized conditions, the proposed sensor exhibited a low limit detection of 0.151 mg/L for nitrite. In addition to high sensitivity, the sensor demonstrated excellent selectivity, good reproducibility, and satisfactory repeatability. The practical applicability of the sensor was evaluated by determining nitrite levels in the of the fish holding tank, yielding good recoveries ranging from 95.02% to 107.93%. Collectively, these results indicate that the Fe_3_O_4_/STAB/CS modified GCE holds great promise for the effective detection of nitrite in real water samples.

## Figures and Tables

**Table 1:** Comparison of various nitrite electrochemical detection sensors.

Analytes	Electrode Material	Technology	Preparation Complexity	Linear Range (mg/L)	Detection Limit (mg/L)	RSD (%)	Reference
Nitrite	[P_2_W_17_V/PEI]_4_/ Fe_3_O_4_@Au/GCE	CV	High (mechanical polishing, aqua regia corrosion, ultrasonic cleaning, electrodeposition, LbL assembly)	69–6900	0.0543	0.73–3.78	[66]
	Fe_3_O_4_@SiO_2_ (acid treated)/modified GCE	DPV	Moderate (acid activation, magnetic separation, washing, redispersion)	1.38–12.42	0.230	0.23–4.92	[53]
	Polydopamine/AuNPs/ SPCE ^a^	ECS ^b^	High (multistep: electrochemical cleaning + Au electrodeposition + activation + pulse-deposition of pDA)	0.69–34.5	0.138	1–7	[67]
	Au-Dy_2_ (WO_4_)_3_/GCE	DPV	Simple (drop-coating of Au-Dy_2_(WO_4_)_3_ + drying + CS capping)	0.69–69	0.2415	-	[68]
	Biofilm sensor	Amperometric	High (two-phase biological cultivation, >30 days)	1–10	0.165	0.82–4.24	[69]
	CTS@Fe_3_O_4_/ SPGNE ^c^	SWV ^d^	Complex (electrolysis washing + two-step ball-milling + three-layer screen-printing with intermediate drying)	0.207–13.8	0.196	-	[70]
	Fe_3_O_4_/STAB/CS/GCE	DPV	Simple (ultrasonic mixing)	0.4–400	0.151	0.91–5.23	This work

Notes: ^a^ screen-printed carbon electrode, ^b^ electrochemical capacitance spectroscopy, ^c^ screen-printed graphene-based electrode, ^d^ square-wave voltametric.

**Table 2:** Detection of nitrite by Fe_3_O_4_/STAB/CS modified GCE in real water.

Samples	Added (mg/L)	Found (mg/L)	Recovery (%)	Relative Standard Deviation (%, *n* = 3)
S_1_	0.6	0.65	107.93	5.23
	1.5	1.47	97.88	5.03
	8	7.59	95.02	3.84
	16	15.31	95.67	4.19
	32	33.29	104.03	1.71
S_2_	0.6	0.63	105.20	0.91
	1.5	1.53	101.97	1.56
	8	8.35	104.33	4.60
	16	15.39	96.21	2.83
	32	32.89	102.79	4.95

## Data Availability

The original contributions presented in this study are included in the article. Further inquiries can be directed to the corresponding author.
